# Diagnostic Accuracy of Clinical and Microbiological Signs in Patients With Skin Lesions Resembling Buruli Ulcer in an Endemic Region

**DOI:** 10.1093/cid/ciy197

**Published:** 2018-04-27

**Authors:** Miriam Eddyani, Ghislain E Sopoh, Gilbert Ayelo, Luc V C Brun, Jean-Jacques Roux, Yves Barogui, Dissou Affolabi, William R Faber, Marleen Boelaert, Annelies Van Rie, Françoise Portaels, Bouke C de Jong

**Affiliations:** 1 Mycobacteriology Unit, Department of Biomedical Sciences, Institute of Tropical Medicine, Antwerp, Belgium; 2 Centre de Dépistage et de Traitement de l’Ulcère de Buruli, Allada; 3 Département d’Anatomie Pathologique, Faculté de Medécine, Université de Parakou, Benin; 4 Centre Hospitalier de Chambéry, France; 5 Centre de Dépistage et de Traitement de l’Ulcère de Buruli, Lalo; 6 Laboratoire de Référence des Mycobactéries, Cotonou, Benin; 7 Academic Medical Centre, Department of Dermatology, University of Amsterdam, The Netherlands; 8 Department of Public Health, Institute of Tropical Medicine, Antwerp, Belgium; 9 Global Health Institute, Department of Epidemiology and Social Medicine, Faculty of Medicine and Health Sciences, University of Antwerp, Belgium

**Keywords:** Buruli ulcer, diagnostic accuracy, diagnosis

## Abstract

**Background:**

The diagnosis of the neglected tropical skin and soft tissue disease Buruli ulcer (BU) is made on clinical and epidemiological grounds, after which treatment with BU-specific antibiotics is initiated empirically. Given the current decline in BU incidence, clinical expertise in the recognition of BU is likely to wane and laboratory confirmation of BU becomes increasingly important. We therefore aimed to determine the diagnostic accuracy of clinical signs and microbiological tests in patients presenting with lesions clinically compatible with BU.

**Methods:**

A total of 227 consecutive patients were recruited in southern Benin and evaluated by clinical diagnosis, direct smear examination (DSE), polymerase chain reaction (PCR), culture, and histopathology. In the absence of a gold standard, the final diagnosis in each patient was made using an expert panel approach. We estimated the accuracy of each test in comparison to the final diagnosis and evaluated the performance of 3 diagnostic algorithms.

**Results:**

Among the 205 patients with complete data, the attending clinicians recognized BU with a sensitivity of 92% (95% confidence interval [CI], 85%–96%), which was higher than the sensitivity of any of the laboratory tests. However, 14% (95% CI, 7%–24%) of patients not suspected to have BU at diagnosis were classified as BU by the expert panel. The specificities of all diagnostics were high (≥91%). All diagnostic algorithms had similar performances.

**Conclusions:**

A broader clinical suspicion should be recommended to reduce missed BU diagnoses. Taking into consideration diagnostic accuracy, time to results, cost-effectiveness, and clinical generalizability, a stepwise diagnostic approach reserving PCR to DSE-negative patients performed best.

## Teaser

A broader clinical suspicion of Buruli ulcer should be recommended to improve accuracy of its clinical diagnosis. Taking into consideration diagnostic accuracy, time to results, cost-effectiveness, and clinical generalizability, a stepwise approach reserving polymerase chain reaction to microscopy-negative patients performed best.

**(See the Editorial Commentary by van der Werf on pages 835–6.)**

Buruli ulcer (BU), a neglected tropical disease caused by *Mycobacterium ulcerans,* is a chronic skin and soft tissue infection that can lead to permanent disfigurement and disability. It is a poorly understood mycobacterial disease, mostly affecting rural populations in West and Central Africa [1, 2].

In most BU-endemic settings, a diagnosis is made on clinical and epidemiological grounds, after which treatment with an 8-week course of rifampicin plus streptomycin or clarithromycin is initiated empirically [3], pending microbiological confirmation where available. BU presents with a diverse range of clinical signs ranging from nodules or edematous plaques to (painless) ulcerations and, rarely, bone involvement. Every clinical expression of BU can be mistaken for another skin condition [4]. Moreover, patients who are not microbiologically confirmed respond better to treatment with antimycobacterial antibiotics, which also cover other bacteria [5].

Buruli ulcer incidence is currently declining in several endemic countries such as Benin, Ivory Coast, and Democratic Republic of Congo. In 2016, 1864 BU patients were reported to the World Health Organization (WHO) by 10 countries, whereas >5000 cases were reported in 2009 [6]. It has been suggested that this decline is, at least in part, due to the introduction of control strategies [6]. The decline in incidence resulted in a decrease in BU lesions relative to non-BU lesions treated in BU facilities [7]. Clinical expertise in the recognition of BU is thus likely to wane, potentially resulting in more diagnostic misclassification. Therefore, laboratory confirmation of BU becomes essential as this would allow improved patient management by initiating more specific therapy in non-BU patients and limiting the prolonged course of rifampicin and streptomycin/clarithromycin to those who truly have BU.

Currently, laboratory diagnostics for BU include culture, direct smear examination (DSE) for acid-fast bacilli, histopathology, and polymerase chain reaction (PCR) targeting the insertion element IS*2404* [8, 9]. Studies on the accuracy of diagnostic tests of BU have used varying reference standards including histopathology [10], PCR [11, 12], at least 1 positive test result [13], a composite reference standard of several tests [12, 14], and latent class analysis (LCA) [15]. This diversity in reference standards makes it difficult to summarize findings on diagnostic test accuracy [8].

The WHO recommends that clinically suspected patients are confirmed by at least 1 of the above-mentioned laboratory tests [16]. Others have proposed a stepwise approach starting with DSE, followed by PCR only in DSE-negative patients to reduce costs [17–19]. Another alternative is a Buruli score based on clinical signs and demographic patient characteristics [15], where only patients with an intermediate score are assessed by PCR.

An accurate diagnostic algorithm that is cost-effective, reduces time to diagnosis, and is feasible in the remote and resource-limited settings where BU is endemic has great potential to reduce misclassification and improve the management of patients presenting with BU-like skin lesions. We therefore aimed to determine the diagnostic accuracy of clinical and microbiological signs in consecutively recruited patients presenting with lesions clinically compatible with BU in a BU-endemic, low-income setting.

## METHODS

### Study Setting and Study Population

Consecutive patients with lesions compatible with BU (ulcers, nodules, edema or plaques) presenting between March 2012 and March 2015 at the Centres de Dépistage et de Traitement de l**’**Ulcère de Buruli (CDTUB) of Allada and Lalo and in 10 health posts of the commune of Zè in southern Benin, living in BU endemic villages, were eligible for study participation. Eligible patients identified at the health posts were referred to the CDTUB for further assessment. Patients presenting with a recent (<2 weeks) wound of obvious noninfectious origin (eg, trauma) or with a wound of >2 weeks with normal healing were excluded from the study.

### Data Collection

All patients were assessed clinically by a triage nurse at the participating CDTUBs or the supervising nurse at the health posts. The nurses’ classification as BU or non-BU based on the WHO diagnostic criteria (young age, residence in an endemic area, undermined edges, location on limbs, necrosis, absence of pain, adenopathy or fever, and hyperpigmented edges) was verified by a clinician, based on a combination of the WHO criteria and clinical experience [3].

Samples for mycobacterial analysis were collected at the CDTUB sites. From each ulcerated lesion, 2 swabs were taken from the undermined edges. From nonulcerative lesions, 2 fine-needle aspirates were taken from the center of the lesion. One sample was processed immediately for DSE after Ziehl-Neelsen (ZN) staining. The second sample was placed in a semisolid transport medium [11], and stored at 4°C until weekly shipment to the Mycobacteriology Reference Laboratory (LRM) in Cotonou, Benin. At the LRM, technicians performed DSE after auramine staining using fluorescence microscopy [20], IS*2404* real-time PCR [21], and in vitro culture for *M. ulcerans* [11, 16].

One 4-mm punch biopsy was taken from every lesion and stored in 10% formalin until embedding in paraffin. One section was stained by hematoxylin-eosin and 1 section by ZN. Histopathological reading was done at the University of Parakou (Benin) and the hospital of Chambéry (France). Both histopathologists were blinded to clinical information (except for age, gender, and type and localization of the lesion) and the results of other diagnostic tests. A standardized reading form was used to score histological changes (Supplementary Data 1). Based on the score, specimens were classified as probable BU (score 7**–**15), compatible with BU (score 4–6), or not compatible with BU (score ≤3). Discordant classifications between the 2 histopathologists were reread and discussed during face-to-face meetings to reach consensus scores that were used in the analysis.

### Data Management

All data were coded and registered in a Microsoft Access database. A dedicated nurse registered all clinical data while a dedicated laboratory technician registered all laboratory data.

A random selection of the DSE readings at both CDTUBs was controlled quarterly by the LRM, which participated in the external quality assurance program for *M. ulcerans* PCR organized by the Antwerp Institute of Tropical Medicine (ITM) [22].

### Data Analysis

#### Index Tests

The accuracy of 6 index tests was evaluated: clinical diagnosis verified by the treating clinician, DSE after ZN staining, DSE after auramine staining, PCR, culture, and histopathology.

#### Reference Standard

Because a gold standard without error or uncertainty is not available for BU [23, 24], the accuracy of each test was estimated using an expert panel approach in the primary analysis [24–26], and PCR, the best currently available diagnostic test, in a secondary analysis [15].

The expert panel approach was based on a stepwise evaluation of all clinical information and laboratory results. The expert panel consisted of 8 study team members and 3 independent dermatologists. The expert panel determined the final diagnosis (status) of each patient in 3 steps. In the first step, patients were classified as confirmed BU (positive by PCR and/or histopathology [score ≥ 7]), possible BU (PCR-negative cases that were positive by DSE, clinically suspected of BU, or had a histopathology score between 4 and 6), or non-BU (negative by all microbiological tests, clinically not suspected of BU and with a histopathology score ≤ 3). In the second step, the expert panel reviewed all patient files classified as possible or non-BU in step 1. Each expert panel member independently made a differential diagnosis for every patient based on all available clinical, demographic, epidemiological, histological and mycobacteriological information (Supplementary Data 2) as well as clinical photographs (missing for 31 patients). In the third step, all cases for whom there was disagreement regarding the classification in step 2 were discussed during 1 of 3 face-to-face meetings until consensus was reached. During these discussions, more weight was given to the opinion of the independent dermatologists compared to those panel members who were also part of the study team. Participants for whom the expert panel failed to reach a consensus were classified as BU.

#### Accuracy of Diagnostic Indicators

The accuracy estimates were calculated as sensitivities, specificities, positive predictive value (PPV), and negative predictive value (NPV). Combined accuracy estimates were also calculated. For histopathology, the accuracy estimates were determined at the cutoff scores for compatible with BU (score 4) and probable BU (score 7) and at the score with an optimal combination of sensitivity and specificity on the receiver operating characteristic (ROC) curve.

The accuracy of 3 diagnostic approaches was then estimated: the WHO recommendation to consider patients who are positive by at least 1 laboratory test as confirmed BU; the stepwise approach to reserve PCR to DSE-negative patients; and the clinical Buruli score that is followed by PCR only when patients have an intermediate score [15] (Supplementary Data 3). Three of the characteristics that make up the Buruli score (yellow and green color, and lesion hyposensitivity) were not available in our dataset. The Buruli score was only tested on ulcerative lesions as 2 of its components (characteristic smell and undermined edges) are unavailable for nonulcerative lesions.

Next, since diagnostic accuracy estimates can vary across patient subgroups, we evaluated effect modification by study site, type of lesion, recruitment type, time since start of study, availability of photographs, patient delay before consultation, transport time of samples to the LRM, and HIV status.

### Statistical Analysis

All analyses were performed in R version 3.3.2. through RStudio version 1.0.136.

The standards for reporting diagnostic studies (STARD [27]) were followed while writing this manuscript (Supplementary Data 14).

### Ethical Considerations

The study protocol was approved by the Provisional National Committee for Ethics in Health Research in Benin, the Institutional Review Board of ITM, and the Ethical Committee of the University Hospital of Antwerp. The study also received administrative authorization of the Benin Ministry of Health Ethics Board. All participants gave written informed consent.

## RESULTS

### Participants

**Figure 1.**
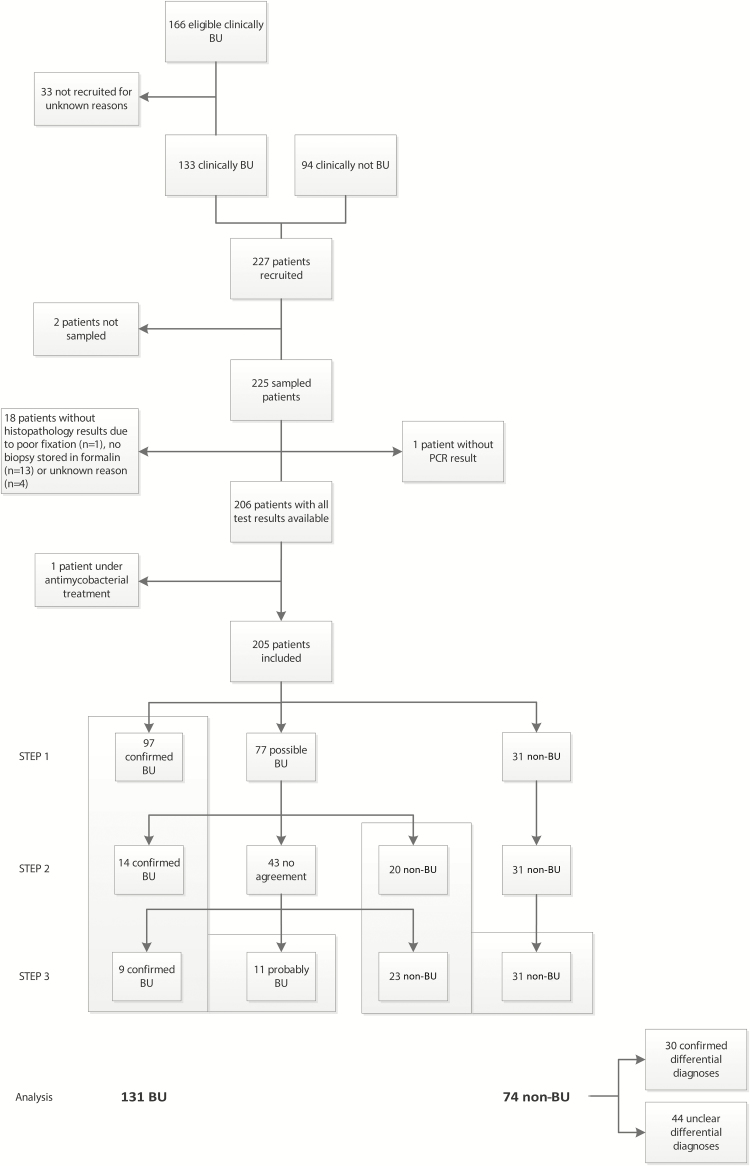
Flow of recruited and included patients, process flow of the participating patients in the 3 steps of the expert panel approach, and the regrouping of confirmed and probable Buruli ulcer (BU) patients during the analysis. In the first step of the expert panel approach, 97 (47%) participants were classified as confirmed BU, 77 (38%) as possible BU, and 31 (15%) as non-BU. The expert panel review (step 2) of the 77 participants with possible BU classified 14 participants as confirmed BU, 20 as non-BU, and 43 with a discordant classification. In the third step, 9 of these 43 participants were classified as confirmed BU and 23 as non-BU. For the remaining 11 participants, the expert panel failed to reach a consensus, and these 11 participants were classified as BU. Abbreviations: BU, Buruli ulcer; PCR, polymerase chain reaction.

Between March 2012 and March 2015, 260 eligible patients presented at the study sites: 166 with symptoms or signs clinically compatible with BU and 94 with lesions clinically not compatible with BU (Figure 1).

Of the 166 patients clinically suspected to have BU, 133 were enrolled. The 33 not recruited did not differ demographically nor clinically from the study participants, suggesting that, at least among those clinically suspected to have BU, there was no overt selection bias (Supplementary Data 4). The total number of eligible patients with lesions clinically not compatible with BU was not documented.

Among the 227 participants recruited, 205 had complete test results and were included in the analysis (Figure 1). Their demographic and clinical characteristics are summarized in Table 1. The patients included in the analysis did not differ demographically nor clinically from the ones with incomplete data (Supplementary Data 5).

**Table 1.** Demographic and Clinical Characteristics of the 205 Study Participants

Characteristic	All Patients		BU Patients (n = 131)		Non-BU Patients (n = 74)		OR	(95% CI)	*P* Value
	Value	(%)	Value	(%)	Value	(%)			
Female sex	89	(43.41)	62	(47.33)	27	(36.49)	1.56	(.87–2.81)	.13
Age, y, median (IQR)	19	(9–42)	12	(9–42)	40	(9–42)			<.00001
CDTUB	113	(55.12)	51	(38.93)	62	(83.78)	0.09	(.04–.18)	<.00001
Allada	100		50		50				
Lalo	13		1		12				
HIV status							0.74	(.17–3.23)	.69
Infected	8	(5.52)	5	(5.00)	3	(6.67)			
Uninfected	137	(94.48)	95	(95.00)	42	(93.33)			
Not tested	60		31		29				
Clinically BU, WHO category	127	(61.95)	120	(91.60)	7	(9.46)	104.42	(38.66–282.04)	<.00001
1	25	(19.69)	22	(18.33)	3	(42.86)			
2	69	(54.33)	65	(54.17)	4	(57.14)			
3	33	(25.98)	33	(27.50)	0				
Clinically non-BU	78	(38.05)	11	(8.40)	67	(90.54)			
Necrotizing fasciitis	36	(46.15)	5	(45.45)	31	(46.27)			
Chronic ulcer	22	(28.21)	2	(18.18)	20	(29.85)			
Abscess	5	(6.41)			5	(7.46)			
Infected wound	3	(3.85)	1	(9.09)	2	(2.99)			
Chronic traumatic wound	2	(2.56)			2	(2.99)			
Tumor	2	(2.56)	1	(9.09)	1	(1.49)			
Erysipelas	1	(1.28)			1	(1.49)			
Osteomyelitis	1	(1.28)	1	(9.09)					
Kaposi sarcoma	1	(1.28)			1	(1.49)			
Varicose ulcer	1	(1.28)			1	(1.49)			
Necrotic wound	1	(1.28)	1	(9.09)					
Suppuration	1	(1.28)			1	(1.49)			
Cervical adenopathy	1	(1.28)			1	(1.49)			
Ganglionary ulcer	1	(1.28)			1	(1.49)			

Data are presented as No. (%) unless otherwise indicated.

Abbreviations: BU, Buruli ulcer; CDTUB, Centres de Dépistage et de Traitement de l**’**Ulcère de Buruli; CI, confidence interval; HIV, human immunodeficiency virus; IQR, interquartile range; OR, odds ratio; WHO, World Health Organization.

### Expert Panel Approach

The expert panel approach classified 131 (64%) patients as BU and 74 (36%) as non-BU (Figure 1). Among the 74 non-BU patients, the expert panel classified 30 as non-BU with confirmed differential diagnoses and 44 as non-BU with unclear differential diagnoses (Supplementary Data 6). Among the 11 patients for whom the expert panel failed to reach a consensus and who were classified as BU, 10 were treated with an 8-week course of rifampicin and streptomycin. One patient was not treated as BU and received ciprofloxacin and cloxacillin.

### Accuracy of Diagnostic Indicators

The results of the index tests are summarized in Supplementary Materials 7 and 8.

**Figure 2.**
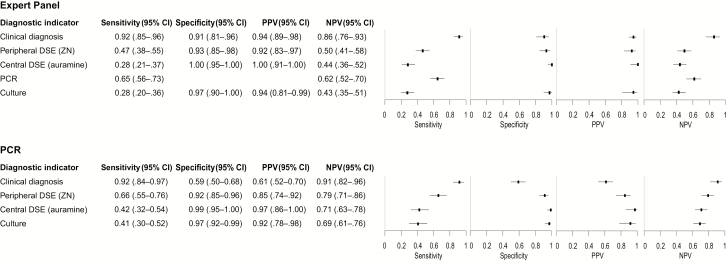
The accuracy estimates of diagnostic indicators with an expert panel and polymerase chain reaction (PCR) as a reference standard. The specificity and positive predictive value of PCR could not be estimated as the expert panel protocol always classified a positive PCR result as a confirmed Buruli ulcer. Abbreviations: CI, confidence interval; DSE, direct smear examination; NPV, negative predictive value; PCR, polymerase chain reaction; PPV, positive predictive value; ZN, Ziehl-Neelsen.

Using the expert panel as the reference, clinical diagnosis by the treating clinician had the highest sensitivity (0.92 [95% confidence interval {CI}, .85–.96]), followed by PCR (0.65 [95% CI, .56–.73]), Ziehl-Neelsen DSE (0.47 [95% CI, .38–.55]), auramine DSE (0.28 [95% CI, .21–.37]), and culture (0.28 [95% CI, .20–.36]) (Figure 2). The specificities and PPV of all diagnostics were high (≥0.91 and ≥0.92, respectively). The NPV of the clinical diagnosis was higher (0.86 [95% CI, .76–.93]) than that of any of the other tests (≤0.62 for all other tests). Using PCR as the reference in a secondary analysis, clinical diagnosis remained the diagnostic method with the highest sensitivity (0.92 [95% CI, .84–.97]) (Figure 2).

For histopathology, the sensitivity and specificity at a cutoff score of 4 (compatible with BU) were 0.54 (95% CI, .45–.63) and 0.73 (95% CI, .61–.83), respectively. At cutoff, 7 (probable BU), the sensitivity and specificity were 0.18 (95% CI, .12–.26) and 1.00 (95% CI, .95–1.00). Using ROC curve analysis, we could not identify a better cutoff given the low combination of sensitivity and specificity across the entire range of scores, with a maximum performance (optimal combination of sensitivity and specificity) at a score of 3.5 (sensitivity: 0.80 [95% CI, .73–.86]; specificity: 0.57 [95% CI, .45–.68]). The area under the curve was 0.73 (95% CI, .67–.80; Supplementary Data 9).

**Figure 3.**
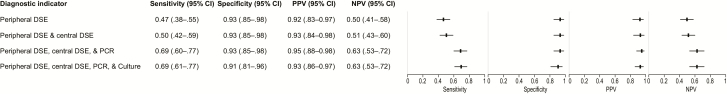
Incremental accuracy estimates. Abbreviations: CI, confidence interval; DSE, direct smear examination; NPV, negative predictive value; PCR, polymerase chain reaction; PPV, positive predictive value.

The accuracy of different combinations of diagnostic tests resulted in an increased sensitivity only when PCR was included without reducing specificity nor PPV (Figure 3).

When evaluating effect modification by important covariates, only the performance of clinical diagnosis differed according to study site and type of lesion, with a lower specificity in decentralized settings, and a higher specificity in ulcerative lesions (Supplementary Data 10). The NPV of both DSE assays, PCR, and culture was lower among patients recruited in decentralized sites, reflecting the higher BU prevalence in decentralized sites.

In a sensitivity analysis, no significant differences were observed in accuracy estimates when classifying the 11 participants for whom the expert panel could not reach a consensus as non-BU (Supplementary Data 11 and 12).

Assuming that sensitivity and specificity of clinical diagnosis and microbiological tests remain constant, a further reduction of the proportion of BU patients would impact the predictive values, with increases in NPV and decreases in PPV (Supplementary Data 13).

### Accuracy of Published Diagnostic Algorithms

**Figure 4.**
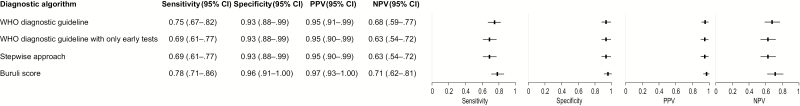
Accuracy estimates of various diagnostic approaches. Abbreviations: CI, confidence interval; NPV, negative predictive value; PPV, positive predictive value; WHO, World Health Organization.

All evaluated diagnostic algorithms had similar performances (Figure 4).

## DISCUSSION

In a BU-endemic setting, trained clinicians clinically diagnose BU with a sensitivity of 92% (95% CI, 85%–96%) and specificity of 91% (95% CI, 81%–96%). The sensitivity of a clinical diagnosis was higher than the sensitivity of any laboratory test. Typical ulcerated BU lesions have indeed been reported to be easily diagnosed clinically in BU-endemic areas by experienced clinicians [16]. Despite the excellent performance of clinical diagnosis, 14% of the study participants clinically not suspected to have BU were reclassified as BU patients by the expert panel procedure, suggesting that there may be a nonnegligible level of underdiagnosis. The majority (64%) of these missed diagnoses were positive by PCR. In clinical practice, these patients do not receive any laboratory test and are not treated for BU unless their clinical evolution suggests BU, resulting in a considerable delay to treatment. However, if the burden of BU would continue reducing, it would virtually never be missed by the clinicians, but a considerable proportion of clinical suspects would not have BU (Supplementary Data 13).

The sensitivity of PCR (65% [95% CI, 56%–73%]) in the present study was lower than generally reported in studies using a variety of reference standards (85.4% [13], 86.0% [28], 87.5% [14], 100% [15]). Clinical characteristics were included into the expert panel approach which resulted in a considerable proportion of PCR-negative patients being classified as BU and therefore more false-negative PCR results than in studies using only microbiological assays as reference standards. However, the addition of PCR to DSE resulted in an increased sensitivity (69% [95% CI, 60%–77%]) while the combined specificity remained high (93% [95% CI, 85%–98%]).

The performance of published diagnostic algorithms for BU in endemic settings was similar. However, the Buruli score—based on clinical and demographic patient characteristics, reserving PCR for patients with an intermediate score [15]—can only be used for the diagnosis of ulcerative lesions. The stepwise approach reserving PCR to DSE-negative patients would therefore be the most cost-effective diagnostic algorithm with the shortest time to results in both ulcerative and nonulcerative lesions.

The performance of histopathology was poor in this study. Its accuracy estimates were low at both cutoff scores of 4 and 7. The ROC analysis of the histopathology consensus scores did not allow the identification of a more optimal cutoff, possibly due to a poor performance of the standardized reading form. Moreover, the pathologists received biopsies in batches blinded to other test results and clinical information. While blinding of histopathologists allows assessing the value of the test per se, it is in contrast with clinical practice where histopathology reading is interpreted taking into account clinical information. Blinding may thus have underestimated the true added value of histopathology, resulting in test review bias [29]. An additional limitation of histopathology in the present analysis is the use of consensus scores. Moreover, the 4-mm punch biopsies recommended by the WHO may sometimes be too small to contain the typical histological changes. Also, the localization of the biopsy in the lesion determines the histological changes captured by the sample. Since in clinical practice histopathology is used when all microbiological tests are negative but there is still a clinical suspicion of BU, a cutoff should be selected at a high sensitivity. However, in the present study the sensitivity of histopathology was disappointingly low at every cutoff.

Because a gold standard without error or uncertainty is not available for BU, we used an expert panel approach. However, the expert panel had access to all available diagnostic indicators, resulting in incorporation bias [23] and possibly overestimating the specificity and PPV of the diagnostic indicators while underestimating their sensitivity and NPV. The true accuracy estimates are probably situated between those measured by PCR and the expert panel. An alternative for diagnostic studies where a good reference standard is lacking is LCA [30], which relates observed patterns of test results to unknown or latent categories of patients—those with and those without the condition. For BU, LCA has only been used in 1 study [15].

Inclusion of multiple sites, clinicians, and histopathologists may have increased interobserver variability. The effect of interobserver variability is likely to be larger for the more subjective tests such as clinical diagnosis and histopathology. We deliberately choose a pragmatic approach, as recommended by Grobbee and Hoes [31], where all diagnostic determinants are assessed as much as possible according to daily practice and by the practicing physician, with some effort to standardize measurements. However, generalizing the study results should be done with care. Health workers in other BU-endemic settings may be less experienced than those working in southern Benin.

## CONCLUSIONS

Despite the excellent performance of clinical diagnosis, there may be an important level of underdiagnosis. A broader clinical suspicion is therefore recommended to reduce missed BU diagnoses allowing improved patient management.

Taking into consideration diagnostic accuracy, time to results, cost-effectiveness, and clinical generalizability, the stepwise diagnostic approach reserving PCR to microscopy-negative patients performed best and would be suitable in the remote and resource-limited settings where BU is endemic.

## Supplementary Data

Supplementary materials are available at *Clinical Infectious Diseases* online. Consisting of data provided by the authors to benefit the reader, the posted materials are not copyedited and are the sole responsibility of the authors, so questions or comments should be addressed to the corresponding author.

Supplementary Material

## Notes

***Acknowledgments.*** We thank Jim Zeegelaar and Felix Atadokpede for their contributions to the clinical expert panel, and Achilleas Tsoumanis, Joris Menten, and Meryam Krit for help with coding in R and advice on statistical analyses.

***Disclaimer.*** The funders had no role in study design, data collection and analysis, decision to publish, or preparation of the manuscript.

***Financial support.*** This work was supported by the Medicor Foundation (www.medicor.li) and UBS Optimus Foundation (www.ubs.com/microsites/optimus-foundation) through the BU differential diagnosis project in Benin. Fondation Follereau Luxembourg (www.ffl.lu/) gives core support to the CDTUB in Allada. M. E. was supported by the Department of Economy, Science and Innovation (www.ewi-vlaanderen.be/) of the Flemish government (Belgium). B. d. J. was supported by the European Research Council-INTERRUPTB (starting grant number 311725).

***Potential conflicts of interest.*** All authors: No reported conflicts of interest. All authors have submitted the ICMJE Form for Disclosure of Potential Conflicts of Interest. Conflicts that the editors consider relevant to the content of the manuscript have been disclosed.
