# Supplementary material for: Diagnostic Accuracy of Clinical and Microbiological Signs in Patients With Skin Lesions Resembling Buruli Ulcer in an Endemic Region
Source: Clin Infect Dis. 2018 Apr 27;67(6):827–34. doi: 10.1093/cid/ciy197 (PMC6117443; doi:10.1093/cid/ciy197)
Supplement: Supplementary Material [file ciy197_suppl_supplementary_material.docx]

**Supplementary material**

**Supplementary material 1.** Reading form for histopathology.

**Supplementary material 2.** Variables made available to the clinical expert panel.

**Supplementary material 3.** Clinical signs and patient demographic characteristics that make up the Buruli score. Three characteristics were not collected in this study.

**Supplementary material 4.** Characteristics of recruited and not recruited patients clinically suspected to have BU.

**Supplementary material 5.** Characteristics of recruited patients that were included or not in the analysis.

**Supplementary material 6.** Differential diagnosis of the 74 non-BU patients.

**Supplementary material 7.** Flow diagram of analyzed patients with the index test results stratified by reference standard.

**Supplementary material 8.** Euler diagram with the number of positive test results among confirmed BU patients.

**Supplementary material 9.** ROC curve of histopathology. The AUC with its 95%CI is plotted as well as the optimal cutoff score with its corresponding specificity and sensitivity.

**Supplementary material 10.** Stratified accuracy estimates (*: significantly different accuracy estimate between the strata).

**Supplementary material 11.** The accuracy estimates of diagnostic indicators (PPV: positive predictive value, NPV: negative predictive value). Possible BU patients are considered non-BU instead of BU.

**Supplementary material 12.** Incremental accuracy estimates (PPV: positive predictive value, NPV: negative predictive value). Possible BU patients are considered non-BU instead of BU.

**Supplementary material 13.** Changing predictive values with varying BU prevalences and fixed values of sensitivity and specificity.

**Supplementary material 14.** STARD (Standards for Reporting of Accuracy Studies) checklist [27].

**Supplementary material 1**. Reading form for histopathology.

| File N°: | **HISTOLOGY RESPONSE FORM : BURULI ULCER** |
| --- | --- |
| Ref CHC : |  |

**READERS** : □ Luc Brun □ JJ Roux □ final form  **Date** .../… /….

| **CLINICAL INFORMATION**: | Age |  | Sex : |  | M |  | F |
| --- | --- | --- | --- | --- | --- | --- | --- |

| Type of lesion : |  | Nodule |  | Plaque |  |  | Edema |  | Ulcer |
| --- | --- | --- | --- | --- | --- | --- | --- | --- | --- |
|  |  | Osteomyelitis |  | Other : |  | | | | |

| Localization of the lesion : |  | URL |  | ULL | | |  |  | LRL |  | LLL |  |
| --- | --- | --- | --- | --- | --- | --- | --- | --- | --- | --- | --- | --- |
|  |  | Head and neck | | |  | Thorax and abdomen | | | |  | buttock and external genital organs | |
|  |  |  |  |  |  |  |  |  |  |  |  |  |

Ongoing treatment : □ No □ Yes duration : ……

Date of sampling :

**QUALITY CONTROL OF SAMPLE**

| **Technical quality** | **0 = Bad**  **5 = Excellent** | | | | | |  |  |  | | | | | |
| --- | --- | --- | --- | --- | --- | --- | --- | --- | --- | --- | --- | --- | --- | --- |
|  | **0** | **1** | **2** | **3** | **4** | **5** |  |  | **0** | **1** | **2** | **3** | **4** | **5** |
| **Fixation** |  |  |  |  |  |  |  | **Ziehl Neelsen** |  |  |  |  |  |  |
| **Orientation** |  |  |  |  |  |  |  | **Gram** |  |  |  |  |  |  |
| **Spreading** |  |  |  |  |  |  |  | **Grocott** |  |  |  |  |  |  |
| **HE Staining** |  |  |  |  |  |  |  | **PAS** |  |  |  |  |  |  |

**Representation of histological layers**

Yes (3 layers present) □

No (1 layer missing) □

Missing layer : □epidermis □dermis □hypodermis

**EPIDERMIS** □Intact □ ulcerated

□Hyperplasia □Psoriasiform □ Other, specify : …………………..

**DERMIS** □ normal □ edema □elastolysis □ necrosis □fibrosis □calcification

□ Hyperplasia of nervous fibers

**HYPODERMIS** Necrosis : □present □absent

Type : □coagulative □ adipose □fibrinoleukocyte □ other : …………….

**CALCIFICATION** : □ yes □no

**HYPERPLASIA OF NERVOUS FIBRES** : □ yes □ no

**INFLAMMATION**

|  | **Epidermis** | **Dermis** | **Hypodermis** |
| --- | --- | --- | --- |
| **Common inflammation** |  |  |  |
| Type (acute, subacute, chronic) |  |  |  |
| Intensity (minimal, moderate, marked) |  |  |  |
| **Specific inflammation** |  |  |  |
| Epitheloid granuloma |  |  |  |
| Predominantly cellular infiltration (eosinophiles, mastocytes, lymphocytes, plasmocytes, histio-monocytes) |  |  |  |

**VESSELS** : □ Vascularitis □Necrosis □ Thrombosis □ thickening

□ Other : specify : ……………………….

**ZIEHL STAINING** :

Presence of AFB □ No

□Yes Intensity : □ minimal (bacilli seen rarely at 40x)

□ Moderate (bacilli in > 10 fields at 40x)

□ Marked (clusters of bacilli seen at 20x)

Localization □epidermis □dermis □hypodermis

□Intracellular □extracellular

**GRAM STAINING** :

□ absence of germs

□ Presence of germs

Type : □ Gram positive cocci □ Gram negative cocci

□ Gram positive bacilli □ Gram negative bacilli

Intensity : □ minimal (cocci/bacilli seen rarely at 40x)

□ Moderate (cocci/bacilli in > 10 fields at 40x)

□ Marked (clusters of cocci/bacilli seen at 20x)

**GROCOTT STAINING** :

□ Absence of fungi

□ Presence of yeasts □ presence of filaments

Intensity : □ minimal (germs rarely seen at 40x)

□ Moderate (germs in > 10 fields at 40x)

□ Marked (clusters of germs seen at 20x)

**PAS STAINING** :

□ Absence of fungi

□ Presence of yeasts □ presence of filaments

Intensity : □ minimal (germs rarely seen at 40x)

□ Moderate (germs in > 10 fields at 40x)

□ Marked (clusters of germs seen at 20x)

□ Other : specify : ……………………………….

**HISTOPATHOLOGICAL SCORE**

| **N°** | **histological signs** | **Score** | | **Remark** |
| --- | --- | --- | --- | --- |
| 1 | Presence of AFB | 5 |  | Reading at 100x with immersion oil ; 1 bacillus is sufficient |
| 2 | Coagulation and/or adipose necrosis  (without active panniculitis) | 4 |  |  |
| 3 | Calcification | 2 |  |  |
| 4 | Tuberculoid granuloma | 2 |  |  |
| 5 | Vascularitis | 1 |  |  |
| 6 | No or minimal inflammation (in the hypodermis) | 1 |  |  |
|  | Histological score | /15 | |  |
| Diagnosis : Probably if score ≥7 ; Compatible if 4 ≤ score ≥ 6; Not compatible if ≤ 3 | | | | |

**CONCLUSION :**

□ HISTOLOGICAL APPEARANCE OF BU

□ INITIAL PHASE □ ACTIVE PHASE □ SCARRING PHASE

□ HISTOLOGICAL APPEARANCE COMPATIBLE WITH BU

□ HISTOLOGICAL APPEARANCE NOT COMPATIBLE WITH BU

Histological appearance rather evoking a pathology of other origin:

□Infectious : □ bacterial □parasitic □fungal

□Vascular

□Tumoral

□Other

Specify suspected diagnosis : ……………………………………………………………………

**Supplementary material 2.** Variables made available to the clinical expert panel.

| **Type of data** | **Indicator** | **Details** |
| --- | --- | --- |
|  | Patient ID | ccccc/xxx/yyyy |
| Clinical | Body weight | Kg |
|  | Body temperature | °C |
|  | Arterial pressure | mmHg |
|  | General condition | bad, weak, slightly good, quite good, good, pale |
|  | WHO category | I, II or III |
|  | Size of lesion | large * small diameter in mm |
|  | Initial clinical diagnosis |  |
|  | Pain (as reported by patient) | yes/no |
|  | Initial lesion (as reported by patient) |  |
|  | Localization of lesion on the body | upper/lower left/right limb, head, buttocks, abdomen, back, thorax, neck |
|  | Type of lesion | ulcer, nodule, plaque, osteomyelitis, edema |
|  | Characteristic BU smell | yes/no |
|  | ulcer base | necrotic, hemorrhagic, clean, granulating exudate, crusty, |
|  | ulcer edges | undermined or not |
|  | Limitation of movement | yes/no |
|  | HIV serology | Positive, negative |
|  | Frequency of bandages |  |
|  | Iron, folic acid and multivitamin supplementation | yes/no |
| Demographic | Age | Years |
|  | Gender | male, female |
|  | Arrondissement |  |
|  | Commune |  |
|  | Village |  |
| Epidemiological | Site of recruitment | CDTUB Allada, CDTUB Lalo or decentralized health post |
|  | Date of referral | dd/mm/yyyy |
|  | Referred by | former patient, teacher, village volunteer, health care worker, self-referral, other |
|  | Reported delay before consultation | days/weeks/months/years |
|  | Reported history of trauma | yes/no |
|  | Residence | endemic/non-endemic region |
|  | Date of tissue sampling | dd/mm/yyyy |
|  | Date of blood sampling | dd/mm/yyyy |
|  | Date of start of BU-specific treatment | dd/mm/yyyy |
|  | Date of excision | dd/mm/yyyy |
|  | Date of end of BU-specific treatment | dd/mm/yyyy |
|  | Outcome | on treatment, healed with/without sequelae, deceased, left against medical advice, referred and lost to follow-up |
| Histopathological | Histopathology score by histopathologist 1 | 0 - 15, including details of score |
|  | Histopathology score by histopathologist 2 | 0 - 15, including details of score |
| Microbiological | Quantification of direct smear examination after ZN staining in laboratory of CDTUB-Allada | negative, scanty, 1+, 2+, 3+ |
|  | Quantification of direct smear examination after auramine staining in laboratory of LRM | negative, scanty, 1+, 2+, 3+ |
|  | IS*2404*-qPCR | Positive, negative |
|  | Interpretation of bacteriology result in terms of responsibility for infection | not, little, quite, very probable |
|  | Identification of bacteriological growth | Species or genus name |
|  | Calcemia | mg/l |
|  | NFS |  |
|  | proteinuria | g/l |

**Supplementary material 3.** Clinical signs and patient demographic characteristics that make up the Buruli score. Three characteristics were not collected in this study.

| **Characteristic** | **Score** |  |
| --- | --- | --- |
| Characteristic smell | +3 |  |
| Yellow color (fibrin) | +2 | Not available |
| Female gender | +2 |  |
| Undermining | +1 |  |
| Green color (pus) | +1 | Not available |
| Lesion hyposensitivity | +1 | Not available |
| Pain at rest | -1 |  |
| Lesion size >5cm | -1 |  |
| Locoregional adenopathy | -2 |  |
| Age >20 and ≤40 years | -3 |  |
| Age >40 years | -5 |  |

| **Supplementary material 4.** Characteristics of recruited and not recruited patients clinically suspected to have BU. | | | | |
| --- | --- | --- | --- | --- |
|  | **Eligible but not recruited (n=33)** | | **Recruited (n=133)** | |
| **Characteristic** | **Value** | **%** | **Value** | **%** |
| Female sex - no. | 17 | 52% | 66 | 50% |
| Age - yr |  |  |  |  |
| Median | 10 |  | 12 |  |
| IQR | 7-22 |  | 8-20 |  |
| Clinically BU - no. | 33 |  | 133 |  |
| WHO Cat. 1 | 4 | 12% | 28 | 21% |
| WHO Cat. 2 | 16 | 48% | 71 | 53% |
| WHO Cat. 3 | 13 | 39% | 34 | 26% |
| Localization of the lesion |  |  |  |  |
| lower limbs | 15 | 42% | 71 | 53% |
| upper limbs | 13 | 36% | 49 | 37% |
| lower and upper limbs | 1 | 3% |  |  |
| other | 4 | 11% | 13 | 10% |

| **Supplementary material 5.** Characteristics of recruited patients that were included or not in the analysis. | | | | |
| --- | --- | --- | --- | --- |
|  | **Not included in analysis (n=22)** | | **Included in analysis (n=205)** | |
| **Characteristic** | **Value** | **%** | **Value** | **%** |
| Female sex - no. | 14 | 64% | 89 | 43% |
| Age - yr |  |  |  |  |
| Median | 15,5 |  | 19 |  |
| IQR | 3-30 |  | 9-42 |  |
| Clinically BU - no. | 6 | 27% | 127 | 62% |
| WHO Cat. 1 | 3 | 50% | 25 | 20% |
| WHO Cat. 2 | 2 | 33% | 69 | 54% |
| WHO Cat. 3 | 1 | 17% | 33 | 26% |
| Localization of the lesion |  |  |  |  |
| lower limbs | 13 | 59% | 136 | 66% |
| upper limbs | 5 | 23% | 50 | 24% |
| other | 4 | 18% | 19 | 9% |
| CDTUB - no. | 12 | 55% | 113 | 55% |
| Allada | 9 |  | 100 |  |
| Lalo | 3 |  | 13 |  |

**Supplementary material 6.** Differential diagnosis of the 74 non-BU patients.

| **Infectious (n=17):** |
| --- |
| necrotizing fasciitis 4 |
| post-abscess ulcer 2 |
| secondary pyoderma 2 |
| cellulitis with ulceration 1 |
| cellulitis 1 |
| Lymphadenitis 1 |
| Mycobacterial infection 1 |
| necrotizing cellulitis 1 |
| nodular lymphangitis 1 |
| Pyoderma 1 |
| secondary infected insect bite 1 |
| Staphylococcal infection 1 |
| tropical ulcer 1 |
|  |
| **Traumatic (n=7):** |
| traumatic ulcer 4 |
| acute post traumatic ulcer 1 |
| post-injection ulcers 1 |
| primary traumatic ulcer 1 |
|  |
| **Tumoral (n=3):** |
| epidermoid cancer 1 |
| metastatic carcinoma 1 |
| Kaposi sarcoma 1 |
|  |
| **Vascular (n=2):** |
| vascular ulcer 2 |
|  |
| **Unknown etiology (n=14):** |
| Non-specific ulcer 9 |
| necrotic ulcer 1 |
| non-healing ulcer of unknown primary cause 1 |
| non-classifiable 2 |
| secondary ulceration 1 |
|  |
| **Undetermined (no photo) (n=30)** |

**Supplementary material 7.** Flow diagram of analyzed patients with the index test results stratified by reference standard.

**Supplementary material 8.** Euler diagram with the number of positive test results among confirmed BU patients.


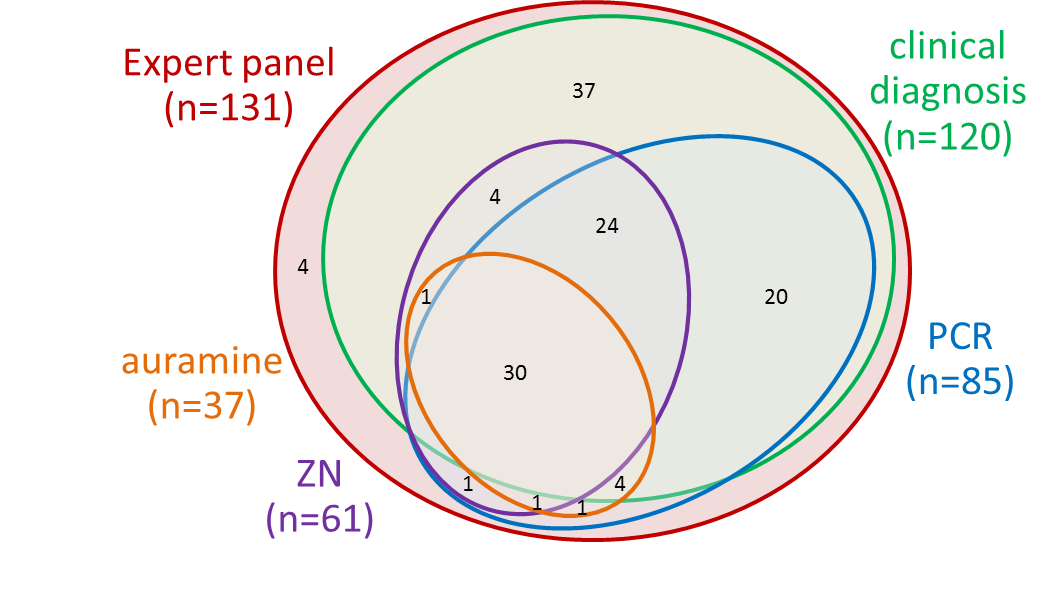


**
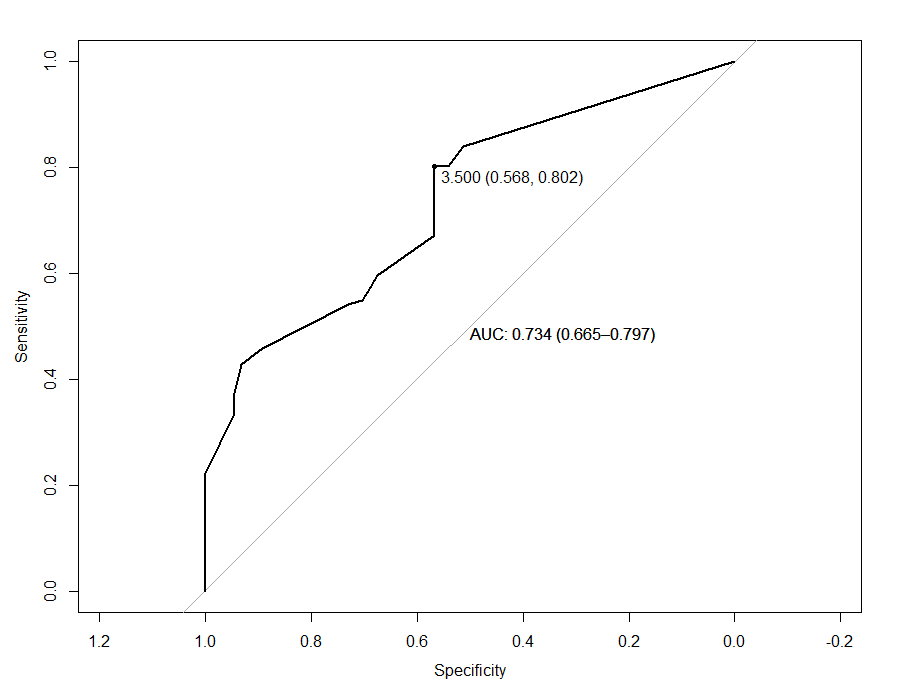
**

**Supplementary material 9.** ROC curve of histopathology. The AUC with its 95%CI is plotted as well as the optimal cutoff score with its corresponding specificity and sensitivity.

**Supplementary material 10.** Stratified accuracy estimates (*: significantly different accuracy estimate between the strata).

|  |  | **Study site** |  |  |  |  |  |  |  |  | | |
| --- | --- | --- | --- | --- | --- | --- | --- | --- | --- | --- | --- | --- |
|  |  | **Decentralised (n=92)** | | | **CDTUB (n=113)** | |  |  |  |  | | |
|  | **test** |  | **95%CI** | |  | **95%CI** | |  | **95%CI** | | | |
| **Sensitivity** | Clinical diagnosis | 0,98 | 0,91 | 1,00 | 0,82 | 0,69 | 0,92 |  |  |  | | |
|  | DSE peripheral | 0,46 | 0,35 | 0,58 | 0,47 | 0,33 | 0,62 |  |  |  | | |
|  | DSE central | 0,24 | 0,15 | 0,35 | 0,35 | 0,22 | 0,50 |  |  |  | | |
|  | PCR | 0,66 | 0,55 | 0,76 | 0,63 | 0,48 | 0,76 |  |  |  | | |
|  | Culture | 0,29 | 0,19 | 0,41 | 0,25 | 0,14 | 0,40 |  |  |  | | |
| **Specificity** | Clinical diagnosis* | 0,50 | 0,21 | 0,79 | 0,98 | 0,91 | 1,00 |  |  |  | | |
|  | DSE peripheral | 0,83 | 0,52 | 0,98 | 0,95 | 0,87 | 0,99 |  |  |  | | |
|  | DSE central | 1,00 | 0,74 | 1,00 | 1,00 | 0,94 | 1,00 |  |  |  | | |
|  | PCR | 1,00 | 0,74 | 1,00 | 1,00 | 0,94 | 1,00 |  |  |  | | |
|  | Culture | 1,00 | 0,74 | 1,00 | 0,96 | 0,88 | 1,00 |  |  |  | | |
| **PPV** | Clinical diagnosis | 0,93 | 0,85 | 0,97 | 0,98 | 0,88 | 1,00 |  |  |  | | |
|  | DSE peripheral | 0,95 | 0,83 | 0,99 | 0,89 | 0,71 | 0,98 |  |  |  | | |
|  | DSE central | 1,00 | 0,82 | 1,00 | 1,00 | 0,81 | 1,00 |  |  |  | | |
|  | PCR | 1,00 | 0,93 | 1,00 | 1,00 | 0,89 | 1,00 |  |  |  | | |
|  | Culture | 1,00 | 0,85 | 1,00 | 0,86 | 0,57 | 0,98 |  |  |  | | |
| **NPV** | Clinical diagnosis | 0,75 | 0,35 | 0,97 | 0,87 | 0,77 | 0,94 |  |  |  | | |
|  | DSE peripheral* | 0,19 | 0,09 | 0,32 | 0,69 | 0,58 | 0,78 |  |  |  | | |
|  | DSE central* | 0,16 | 0,09 | 0,27 | 0,65 | 0,55 | 0,75 |  |  |  | | |
|  | PCR* | 0,31 | 0,17 | 0,48 | 0,77 | 0,66 | 0,85 |  |  |  | | |
|  | Culture* | 0,18 | 0,10 | 0,30 | 0,60 | 0,49 | 0,70 |  |  |  | | |
|  |  | **Type of lesion** | |  |  |  |  |  |  |  | | |
|  |  | **Ulcerative (n=187)** | |  | **Nonulcerative (n=18)** | | |  |  |  | | |
| **Sensitivity** | Clinical diagnosis | 0,91 | 0,84 | 0,95 | 1,00 | 0,74 | 1,00 |  |  |  | | |
|  | DSE peripheral | 0,48 | 0,39 | 0,57 | 0,33 | 0,10 | 0,65 |  |  |  | | |
|  | DSE central | 0,29 | 0,21 | 0,38 | 0,17 | 0,02 | 0,48 |  |  |  | | |
|  | PCR | 0,65 | 0,55 | 0,73 | 0,67 | 0,35 | 0,90 |  |  |  | | |
|  | Culture | 0,27 | 0,19 | 0,36 | 0,33 | 0,10 | 0,65 |  |  |  | | |
| **Specificity** | Clinical diagnosis* | 0,96 | 0,88 | 0,99 | 0,33 | 0,04 | 0,78 |  |  |  | | |
|  | DSE peripheral | 0,94 | 0,86 | 0,98 | 0,83 | 0,36 | 1,00 |  |  |  | | |
|  | DSE central | 1,00 | 0,95 | 1,00 | 1,00 | 0,54 | 1,00 |  |  |  | | |
|  | PCR | 1,00 | 0,95 | 1,00 | 1,00 | 0,54 | 1,00 |  |  |  | | |
|  | Cultureure | 0,97 | 0,89 | 1,00 | 1,00 | 0,48 | 1,00 |  |  |  | | |
| **PPV** | Clinical diagnosis* | 0,97 | 0,92 | 0,99 | 0,75 | 0,48 | 0,93 |  |  |  | | |
|  | DSE peripheral | 0,93 | 0,84 | 0,98 | 0,80 | 0,28 | 0,99 |  |  |  | | |
|  | DSE central | 1,00 | 0,90 | 1,00 | 1,00 | 0,16 | 1,00 |  |  |  | | |
|  | PCR | 1,00 | 0,95 | 1,00 | 1,00 | 0,63 | 1,00 |  |  |  | | |
|  | Culture | 0,94 | 0,79 | 0,99 | 1,00 | 0,40 | 1,00 |  |  |  | | |
| **NPV** | Clinical diagnosis | 0,86 | 0,76 | 0,93 | 1,00 | 0,16 | 1,00 |  |  |  | | |
|  | DSE peripheral | 0,51 | 0,42 | 0,60 | 0,38 | 0,14 | 0,68 |  |  |  | | |
|  | DSE central | 0,45 | 0,37 | 0,53 | 0,38 | 0,15 | 0,65 |  |  |  | | |
|  | PCR | 0,62 | 0,52 | 0,71 | 0,60 | 0,26 | 0,88 |  |  |  | | |
|  | Culture | 0,43 | 0,35 | 0,52 | 0,38 | 0,14 | 0,68 |  |  |  | | |
|  |  | **Recruitment type** | |  |  |  |  |  |  |  | | |
|  |  | **Active case-finding (n=143)** | | | **Passive case-finding (n=62)** | | | |  |  | | |
| **Sensitivity** | Clinical diagnosis | 0,93 | 0,86 | 0,97 | 0,87 | 0,69 | 0,96 |  |  |  | | |
|  | DSE peripheral | 0,48 | 0,37 | 0,58 | 0,43 | 0,25 | 0,63 |  |  |  | | |
|  | DSE central | 0,27 | 0,18 | 0,36 | 0,33 | 0,17 | 0,53 |  |  |  | | |
|  | PCR | 0,67 | 0,57 | 0,76 | 0,57 | 0,37 | 0,75 |  |  |  | | |
|  | Culture | 0,28 | 0,20 | 0,39 | 0,25 | 0,11 | 0,45 |  |  |  | | |
| **Specificity** | Clinical diagnosis | 0,83 | 0,69 | 0,93 | 1,00 | 0,89 | 1,00 |  |  |  | | |
|  | DSE peripheral | 0,95 | 0,84 | 0,99 | 0,91 | 0,75 | 0,98 |  |  |  | | |
|  | DSE central | 1,00 | 0,92 | 1,00 | 1,00 | 0,89 | 1,00 |  |  |  | | |
|  | PCR | 1,00 | 0,92 | 1,00 | 1,00 | 0,89 | 1,00 |  |  |  | | |
|  | Culture | 0,97 | 0,86 | 1,00 | 0,97 | 0,83 | 1,00 |  |  |  | | |
| **PPV** | Clinical diagnosis | 0,93 | 0,86 | 0,97 | 1,00 | 0,87 | 1,00 |  |  |  | | |
|  | DSE peripheral | 0,96 | 0,86 | 1,00 | 0,81 | 0,54 | 0,96 |  |  |  | | |
|  | DSE central | 1,00 | 0,87 | 1,00 | 1,00 | 0,69 | 1,00 |  |  |  | | |
|  | PCR | 1,00 | 0,95 | 1,00 | 1,00 | 0,80 | 1,00 |  |  |  | | |
|  | Culture | 0,96 | 0,82 | 1,00 | 0,88 | 0,47 | 1,00 |  |  |  | | |
| **NPV** | Clinical diagnosis | 0,83 | 0,69 | 0,93 | 0,89 | 0,74 | 0,97 |  |  |  | | |
|  | DSE peripheral | 0,43 | 0,33 | 0,54 | 0,63 | 0,48 | 0,77 |  |  |  | | |
|  | DSE central* | 0,36 | 0,27 | 0,46 | 0,62 | 0,47 | 0,75 |  |  |  | | |
|  | PCR | 0,56 | 0,44 | 0,67 | 0,71 | 0,56 | 0,84 |  |  |  | | |
|  | Culture | 0,35 | 0,26 | 0,45 | 0,58 | 0,43 | 0,72 |  |  |  | | |
|  |  | **HIV status** |  |  |  |  |  |  |  |  | | |
|  |  | **Positive (n=8)** | |  | **Negative (n=137)** | | |  |  |  | | |
| **Sensitivity** | Clinical diagnosis | 0,80 | 0,28 | 0,99 | 0,92 | 0,84 | 0,96 |  |  |  | | |
|  | DSE peripheral | 0,80 | 0,28 | 0,99 | 0,47 | 0,37 | 0,58 |  |  |  | | |
|  | DSE central | 0,80 | 0,28 | 0,99 | 0,28 | 0,20 | 0,39 |  |  |  | | |
|  | PCR | 1,00 | 0,48 | 1,00 | 0,64 | 0,54 | 0,74 |  |  |  | | |
|  | Culture | 0,40 | 0,05 | 0,85 | 0,29 | 0,20 | 0,39 |  |  |  | | |
| **Specificity** | Clinical diagnosis | 1,00 | 0,29 | 1,00 | 0,86 | 0,71 | 0,95 |  |  |  | | |
|  | DSE peripheral | 1,00 | 0,29 | 1,00 | 0,88 | 0,74 | 0,96 |  |  |  | | |
|  | DSE central | 1,00 | 0,29 | 1,00 | 1,00 | 0,92 | 1,00 |  |  |  | | |
|  | PCR | 1,00 | 0,29 | 1,00 | 1,00 | 0,92 | 1,00 |  |  |  | | |
|  | Culture | 1,00 | 0,29 | 1,00 | 0,97 | 0,86 | 1,00 |  |  |  | | |
| **PPV** | Clinical diagnosis | 1,00 | 0,40 | 1,00 | 0,94 | 0,86 | 0,98 |  |  |  | | |
|  | DSE peripheral | 1,00 | 0,40 | 1,00 | 0,90 | 0,78 | 0,97 |  |  |  | | |
|  | DSE central | 1,00 | 0,40 | 1,00 | 1,00 | 0,87 | 1,00 |  |  |  | | |
|  | PCR | 1,00 | 0,48 | 1,00 | 1,00 | 0,94 | 1,00 |  |  |  | | |
|  | Culture | 1,00 | 0,16 | 1,00 | 0,96 | 0,81 | 1,00 |  |  |  | | |
| **NPV** | Clinical diagnosis | 0,75 | 0,19 | 0,99 | 0,82 | 0,67 | 0,92 |  |  |  | | |
|  | DSE peripheral | 0,75 | 0,19 | 0,99 | 0,43 | 0,32 | 0,54 |  |  |  | | |
|  | DSE central | 0,75 | 0,19 | 0,99 | 0,38 | 0,29 | 0,48 |  |  |  | | |
|  | PCR | 1,00 | 0,29 | 1,00 | 0,55 | 0,43 | 0,67 |  |  |  | | |
|  | Culture | 0,50 | 0,12 | 0,88 | 0,36 | 0,27 | 0,46 |  |  |  | | |
|  |  | **Availability of photographs to clinical expert panel** | | | | | |  |  |  | | |
|  |  | **Yes (n=72)** |  |  | **No (n=31)** | |  |  |  |  | | |
| **Sensitivity** | Clinical diagnosis | 0,90 | 0,73 | 0,98 | 1,00 | 0,54 | 1,00 |  |  | |  | |
|  | DSE peripheral | 0,07 | 0,01 | 0,22 | 0,33 | 0,04 | 0,78 |  |  | |  | |
|  | DSE central | 0,00 | 0,00 | 0,12 | 0,00 | 0,00 | 0,46 |  |  | |  | |
|  | PCR | 0,00 | 0,00 | 0,12 | 0,00 | 0,00 | 0,46 |  |  | |  | |
|  | Culture | 0,00 | 0,00 | 0,12 | 0,00 | 0,00 | 0,52 |  |  | |  | |
| **Specificity** | Clinical diagnosis | 0,86 | 0,71 | 0,95 | 1,00 | 0,86 | 1,00 |  |  | |  | |
|  | DSE peripheral | 0,93 | 0,81 | 0,99 | 0,96 | 0,80 | 1,00 |  |  | |  | |
|  | DSE central | 1,00 | 0,92 | 1,00 | 1,00 | 0,86 | 1,00 |  |  | |  | |
|  | PCR | 1,00 | 0,92 | 1,00 | 1,00 | 0,86 | 1,00 |  |  | |  | |
|  | Culture | 1,00 | 0,91 | 1,00 | 0,91 | 0,72 | 0,99 |  |  | |  | |
| **PPV** | Clinical diagnosis | 0,82 | 0,65 | 0,93 | 1,00 | 0,54 | 1,00 |  |  | |  | |
|  | DSE peripheral | 0,40 | 0,05 | 0,85 | 0,67 | 0,09 | 0,99 |  |  | |  | |
|  | DSE central | NA | 0,00 | 1,00 | NA | 0,00 | 1,00 |  |  | |  | |
|  | PCR | NA | 0,00 | 1,00 | NA | 0,00 | 1,00 |  |  | |  | |
|  | Culture | NA | 0,00 | 1,00 | 0,00 | 0,00 | 0,84 |  |  | |  | |
| **NPV** | Clinical diagnosis | 0,92 | 0,79 | 0,98 | 1,00 | 0,86 | 1,00 |  |  | |  | |
|  | DSE peripheral | 0,58 | 0,46 | 0,70 | 0,86 | 0,67 | 0,96 |  |  | |  | |
|  | DSE central | 0,58 | 0,46 | 0,70 | 0,81 | 0,63 | 0,93 |  |  | |  | |
|  | PCR | 0,58 | 0,46 | 0,70 | 0,81 | 0,63 | 0,93 |  |  | |  | |
|  | Culture | 0,58 | 0,46 | 0,70 | 0,81 | 0,61 | 0,93 |  |  | |  | |
|  |  | **Time after start of the study** | | |  |  |  |  |  | |  | |
|  |  | **Year 1 (n=71)** | |  | **Year 2 (n=98)** | |  | **Year 3 (n=36)** | | | |  |
| **Sensitivity** | Clinical diagnosis | 0,91 | 0,78 | 0,97 | 0,90 | 0,79 | 0,96 | 0,97 | 0,82 | | 1,00 | |
|  | DSE peripheral | 0,48 | 0,32 | 0,63 | 0,40 | 0,27 | 0,53 | 0,59 | 0,39 | | 0,76 | |
|  | DSE central | 0,23 | 0,11 | 0,38 | 0,26 | 0,15 | 0,39 | 0,41 | 0,24 | | 0,61 | |
|  | PCR | 0,68 | 0,52 | 0,81 | 0,59 | 0,45 | 0,71 | 0,72 | 0,53 | | 0,87 | |
|  | Culture | 0,19 | 0,09 | 0,34 | 0,30 | 0,18 | 0,44 | 0,36 | 0,19 | | 0,56 | |
| **Specificity** | Clinical diagnosis | 0,89 | 0,71 | 0,98 | 0,93 | 0,80 | 0,98 | 0,86 | 0,42 | | 1,00 | |
|  | DSE peripheral | 0,89 | 0,71 | 0,98 | 1,00 | 0,91 | 1,00 | 0,71 | 0,29 | | 0,96 | |
|  | DSE central | 1,00 | 0,87 | 1,00 | 1,00 | 0,91 | 1,00 | 1,00 | 0,59 | | 1,00 | |
|  | PCR | 1,00 | 0,87 | 1,00 | 1,00 | 0,91 | 1,00 | 1,00 | 0,59 | | 1,00 | |
|  | Culture | 1,00 | 0,87 | 1,00 | 0,94 | 0,81 | 0,99 | 1,00 | 0,59 | | 1,00 | |
| **PPV** | Clinical diagnosis | 0,93 | 0,81 | 0,99 | 0,95 | 0,85 | 0,99 | 0,97 | 0,82 | | 1,00 | |
|  | DSE peripheral | 0,88 | 0,68 | 0,97 | 1,00 | 0,85 | 1,00 | 0,89 | 0,67 | | 0,99 | |
|  | DSE central | 1,00 | 0,69 | 1,00 | 1,00 | 0,78 | 1,00 | 1,00 | 0,74 | | 1,00 | |
|  | PCR | 1,00 | 0,88 | 1,00 | 1,00 | 0,90 | 1,00 | 1,00 | 0,84 | | 1,00 | |
|  | Culture | 1,00 | 0,63 | 1,00 | 0,89 | 0,65 | 0,99 | 1,00 | 0,69 | | 1,00 | |
| **NPV** | Clinical diagnosis | 0,86 | 0,67 | 0,96 | 0,86 | 0,72 | 0,95 | 0,86 | 0,42 | | 1,00 | |
|  | DSE peripheral | 0,51 | 0,36 | 0,66 | 0,53 | 0,41 | 0,65 | 0,29 | 0,10 | | 0,56 | |
|  | DSE central | 0,44 | 0,32 | 0,58 | 0,48 | 0,37 | 0,59 | 0,29 | 0,13 | | 0,51 | |
|  | PCR | 0,66 | 0,49 | 0,80 | 0,63 | 0,50 | 0,74 | 0,47 | 0,21 | | 0,73 | |
|  | Culture | 0,43 | 0,31 | 0,57 | 0,47 | 0,35 | 0,59 | 0,28 | 0,12 | | 0,49 | |
|  |  | **Reported delay before first consultation** | | | | |  |  |  | |  | |
|  |  | **=<3 months (n=175)** | |  | **>3 months (n=30)** | | |  |  | |  | |
| **Sensitivity** | Clinical diagnosis | 0,91 | 0,84 | 0,96 | 0,95 | 0,74 | 1,00 |  |  | |  | |
|  | DSE peripheral | 0,48 | 0,39 | 0,58 | 0,37 | 0,16 | 0,62 |  |  | |  | |
|  | DSE central | 0,29 | 0,21 | 0,39 | 0,21 | 0,06 | 0,46 |  |  | |  | |
|  | PCR | 0,65 | 0,56 | 0,74 | 0,63 | 0,38 | 0,84 |  |  | |  | |
|  | Culture | 0,28 | 0,19 | 0,37 | 0,28 | 0,10 | 0,53 |  |  | |  | |
| **Specificity** | Clinical diagnosis | 0,92 | 0,82 | 0,97 | 0,82 | 0,48 | 0,98 |  |  | |  | |
|  | DSE peripheral | 0,97 | 0,89 | 1,00 | 0,73 | 0,39 | 0,94 |  |  | |  | |
|  | DSE central | 1,00 | 0,94 | 1,00 | 1,00 | 0,72 | 1,00 |  |  | |  | |
|  | PCR | 1,00 | 0,94 | 1,00 | 1,00 | 0,72 | 1,00 |  |  | |  | |
|  | Culture | 0,97 | 0,88 | 1,00 | 1,00 | 0,69 | 1,00 |  |  | |  | |
| **PPV** | Clinical diagnosis | 0,95 | 0,89 | 0,98 | 0,90 | 0,68 | 0,99 |  |  | |  | |
|  | DSE peripheral | 0,96 | 0,88 | 1,00 | 0,70 | 0,35 | 0,93 |  |  | |  | |
|  | DSE central | 1,00 | 0,89 | 1,00 | 1,00 | 0,40 | 1,00 |  |  | |  | |
|  | PCR | 1,00 | 0,95 | 1,00 | 1,00 | 0,74 | 1,00 |  |  | |  | |
|  | Culture | 0,94 | 0,79 | 0,99 | 1,00 | 0,48 | 1,00 |  |  | |  | |
| **NPV** | Clinical diagnosis | 0,85 | 0,75 | 0,93 | 0,90 | 0,55 | 1,00 |  |  | |  | |
|  | DSE peripheral | 0,51 | 0,42 | 0,61 | 0,40 | 0,19 | 0,64 |  |  | |  | |
|  | DSE central | 0,44 | 0,36 | 0,53 | 0,42 | 0,23 | 0,63 |  |  | |  | |
|  | PCR | 0,62 | 0,52 | 0,71 | 0,61 | 0,36 | 0,83 |  |  | |  | |
|  | Culture | 0,42 | 0,34 | 0,51 | 0,43 | 0,23 | 0,66 |  |  | |  | |
|  |  | **Transport time to the laboratory** | | | |  |  |  |  | |  | |
|  |  | **=<7 days (n=106)** | |  | **>7 days (n=99)** | | |  |  | |  | |
| **Sensitivity** | Clinical diagnosis | 0,94 | 0,85 | 0,98 | 0,89 | 0,79 | 0,95 |  |  | |  | |
|  | DSE peripheral | 0,49 | 0,37 | 0,62 | 0,44 | 0,31 | 0,57 |  |  | |  | |
|  | DSE central | 0,27 | 0,17 | 0,39 | 0,30 | 0,19 | 0,42 |  |  | |  | |
|  | PCR | 0,63 | 0,50 | 0,74 | 0,67 | 0,54 | 0,78 |  |  | |  | |
|  | Culture | 0,32 | 0,21 | 0,45 | 0,22 | 0,13 | 0,35 |  |  | |  | |
| **Specificity** | Clinical diagnosis | 0,87 | 0,73 | 0,96 | 0,94 | 0,81 | 0,99 |  |  | |  | |
|  | DSE peripheral | 0,90 | 0,76 | 0,97 | 0,97 | 0,85 | 1,00 |  |  | |  | |
|  | DSE central | 1,00 | 0,91 | 1,00 | 1,00 | 0,90 | 1,00 |  |  | |  | |
|  | PCR | 1,00 | 0,91 | 1,00 | 1,00 | 0,90 | 1,00 |  |  | |  | |
|  | Culture | 1,00 | 0,90 | 1,00 | 0,94 | 0,80 | 0,99 |  |  | |  | |
| **PPV** | Clinical diagnosis | 0,93 | 0,84 | 0,98 | 0,97 | 0,88 | 1,00 |  |  | |  | |
|  | DSE peripheral | 0,89 | 0,75 | 0,97 | 0,97 | 0,82 | 1,00 |  |  | |  | |
|  | DSE central | 1,00 | 0,81 | 1,00 | 1,00 | 0,82 | 1,00 |  |  | |  | |
|  | PCR | 1,00 | 0,92 | 1,00 | 1,00 | 0,92 | 1,00 |  |  | |  | |
|  | Culture | 1,00 | 0,84 | 1,00 | 0,87 | 0,60 | 0,98 |  |  | |  | |
| **NPV** | Clinical diagnosis | 0,89 | 0,75 | 0,97 | 0,83 | 0,67 | 0,93 |  |  | |  | |
|  | DSE peripheral | 0,51 | 0,38 | 0,63 | 0,49 | 0,36 | 0,61 |  |  | |  | |
|  | DSE central | 0,44 | 0,34 | 0,55 | 0,44 | 0,33 | 0,55 |  |  | |  | |
|  | PCR | 0,61 | 0,48 | 0,73 | 0,63 | 0,49 | 0,75 |  |  | |  | |
|  | Culture | 0,44 | 0,33 | 0,56 | 0,41 | 0,30 | 0,53 |  |  | |  | |

**Supplementary material 11.** The accuracy estimates of diagnostic indicators (PPV: positive predictive value, NPV: negative predictive value). Probable BU patients are considered non-BU instead of BU.

| **Diagnostic indicator** | **Sensitivity** | **95% CI** | | **Specificity** | **95% CI** | | **PPV** | **95% CI** | | **NPV** | **95% CI** | |
| --- | --- | --- | --- | --- | --- | --- | --- | --- | --- | --- | --- | --- |
| Clinical diagnosis | 0.92 | 0.85 | 0.96 | 0.80 | 0.70 | 0.88 | 0.87 | 0.79 | 0.92 | 0.87 | 0.78 | 0.94 |
| DSE after ZN staining | 0.51 | 0.42 | 0.60 | 0.94 | 0.87 | 0.98 | 0.92 | 0.83 | 0.97 | 0.58 | 0.49 | 0.66 |
| DSE after auramine staining | 0.31 | 0.23 | 0.40 | 1.00 | 0.96 | 1.00 | 1.00 | 0.91 | 1.00 | 0.51 | 0.43 | 0.58 |
| PCR | 0.71 | 0.62 | 0.79 |  |  |  |  |  |  | 0.71 | 0.62 | 0.79 |
| Culture | 0.30 | 0.22 | 0.40 | 0.97 | 0.91 | 1.00 | 0.94 | 0.81 | 0.99 | 0.50 | 0.42 | 0.58 |

**Supplementary material 12.** Incremental accuracy estimates (PPV: positive predictive value, NPV: negative predictive value). Possible BU patients are considered non-BU instead of BU.

| **Diagnostic indicator** | **Sensitivity** | **95% CI** | | **Specificity** | **95% CI** | | **PPV** | **95% CI** | | **NPV** | **95% CI** | |
| --- | --- | --- | --- | --- | --- | --- | --- | --- | --- | --- | --- | --- |
| DSE_peripheral_ | 0.51 | 0.42 | 0.60 | 0.94 | 0.87 | 0.98 | 0.92 | 0.83 | 0.97 | 0.58 | 0.49 | 0.66 |
| DSE_peripheral_ & DSE_central_ | 0,55 | 0,46 | 0,64 | 0,94 | 0,87 | 0,98 | 0,93 | 0,84 | 0,98 | 0,60 | 0,51 | 0,68 |
| DSE_peripheral_ & DSE_central_ & PCR | 0,75 | 0,66 | 0,82 | 0,94 | 0,87 | 0,98 | 0,95 | 0,88 | 0,98 | 0,73 | 0,63 | 0,81 |
| DSE_peripheral_ & DSE_central_ & PCR & Culture | 0,76 | 0,67 | 0,83 | 0,92 | 0,84 | 0,97 | 0,93 | 0,86 | 0,97 | 0,73 | 0,63 | 0,81 |

**Supplementary material 13.** Changing predictive values with varying BU prevalences and fixed values of sensitivity and specificity.

**Supplementary material 14.** STARD (Standards for Reporting of Accuracy Studies) checklist [27].

| **Section and topic** | **No** | **Item** | **Details** |
| --- | --- | --- | --- |
| **Title or abstract** | | | |
|  | 1 | Identification as a study of diagnostic accuracy using at least one measure of accuracy (such as sensitivity, specificity, predictive values or AUC) | Title: Diagnostic accuracy of clinical and microbiological signs of Buruli ulcer in patients with skin lesions in an endemic region |
| **Abstract** | | | |
|  | 2 | Structured summary of study design, methods, results and conclusions | See abstract |
| **Introduction** | | | |
|  | 3 | Scientific and clinical background, including the intended use and clinical role of the index test | See introduction |
|  | 4 | Study objectives and hypotheses | We aimed to determine the diagnostic accuracy of clinical and microbiological signs of BU in consecutively recruited patients presenting with lesions clinically compatible with BU in a BU-endemic, low-income setting. |
| **Methods** | | | |
| Study design | 5 | Whether data collection was planned before the index test and reference standard were performed (prospective study) or after (retrospective study) | Patients were recruited consecutively and data collected prospectively. |
| Participants | 6 | Eligibility criteria | Patients presenting with a recent (<2 weeks old) wound of an obvious non-infectious origin (e.g. trauma) or with a chronic (>2 weeks) wound with a normal healing process were excluded from the study. |
|  | 7 | On what basis potentially eligible participants were identified (such as symptoms, results from previous tests, inclusion in registry) | Patients with skin lesions compatible with BU (ulcers, nodules, edema or plaques) were eligible for study participation. |
|  | 8 | Where and when potentially eligible participants were identified (setting, location and dates) | Patients presenting between March 2012 and March 2015 for care at the Centres de Dépistage et de Traitement de l’Ulcère de Buruli (CDTUB) of Allada and Lalo and in ten health posts of the commune of Zè (supervised by the CDTUB of Allada) in southern Benin, were eligible for study participation. |
|  | 9 | Whether participants formed a consecutive, random or convenience series | Patients were recruited consecutively. |
| Test methods | 10a | Index test, in sufficient detail to allow replication | See methods |
|  | 10b | Reference standard, in sufficient detail to allow replication | See methods |
|  | 11 | Rationale for choosing the reference standard (if alternatives exist) | Because a gold standard without error or uncertainty is not available for BU, the accuracy of each test was estimated using an expert panel approach in the primary analysis, and PCR, the best currently available diagnostic, in a secondary analysis. |
|  | 12a | Definition of and rationale for test positivity cut-offs or result categories of the index test, distinguishing prespecified from exploratory | See methods for histopathology scoring (see supplementary information 1) |
|  | 12b | Definition of and rationale for test positivity cut-offs or result categories of the reference standard, distinguishing prespecified from exploratory | See methods for expert panel approach |
|  | 13a | Whether clinical information and reference standard results were available to the performers or readers of the index test | Laboratory test results were not (yet) available to the clinical team at time of clinical diagnosis. Both histopathologists were blinded to clinical information (except for age, gender, type and localization of the lesion) and the results of other diagnostic tests. |
|  | 13b | Whether clinical information and index test results were available to the assessors of the reference standard | The expert panel making the final classification of patients used all available clinical, demographic, epidemiological, histological and mycobacterial information (see supplementary material 2) |
| Analysis | 14 | Methods for estimating or comparing measures of diagnostic accuracy | See methods |
|  | 15 | How indeterminate index test or reference standard results were handled | Participants for whom the expert panel failed to reach a consensus were classified as ‘BU’ because they would be managed as BU patients in clinical practice. |
|  | 16 | How missing data on the index test and reference standard were handled | Only the 205 patients with complete data were included in the analysis (see fig 1). |
|  | 17 | Any analyses of variability in diagnostic accuracy, distinguishing prespecified from exploratory | Since diagnostic accuracy estimates can vary across patient subgroups, we evaluated effect modification by study site, type of lesion, recruitment type, time since start of study, availability of photographs to the expert panel, patient delay before consultation, transport time of samples to the laboratory in Cotonou, and HIV status. |
|  | 18 | Intended sample size and how it was determined | Not specified in manuscript: The number of patients suspected to have BU needed to estimate the sensitivity of the clinical diagnosis at 85% with a precision of 6.5% was estimated. A sample size of 116 « true » BU patients was needed.  According to the health statistics at the start of the study, 50% of suspected cases at the CDTUB were clinically diagnosed to have BU. Among these clinically diagnosed patients, 55% were confirmed by PCR (positive predictive value). Moreover, we assumed that among the patients who were clinically declared not to have BU, 95% were negative by PCR (negative predictive value). We then estimated a prevalence of PCR-confirmed BU of 30% (50%*55% + 50%*5%) among patients suspected to have BU. With a PCR confirmed BU prevalence of 30%, this results in a sample size estimate of patients suspected to have BU of 387 (116 / 30%).  According to the reports of the national program , the proportion of BU patients lost-to-follow- up was 2%. Assuming a refusal to give informed consent at the same proportion, we foresee a number of 15 (387*4%) refusals or lost-to-follow-up, bringing the final sample size estimate at 402 (among whom 201 BU and 201 non-BU). During the recruitment period the incidence of Buruli ulcer in Benin reduced more than we had expected. We were therefore obliged to continue recruiting 2 years longer than initially foreseen, while still not reaching the anticipated sample size of 402 patients. We calculated that a reduction in the sample size to 240 patients would have a limited impact on the precision of estimates (the precision of the confidence interval around the sensitivity estimate would reduce from 6.6% to 7.8%). |
| **Results** | | | |
| Participants | 19 | Flow of participants, using a diagram | See figure 1 and supplementary material 7 |
|  | 20 | Baseline demographic and clinical characteristics of participants | See table 1 |
|  | 21a | Distribution of severity of disease in those with the target condition | See table 1 |
|  | 21b | Distribution of alternative diagnoses in those without the target condition | See supplementary material 6 |
|  | 22 | Time interval and any clinical interventions between index test and reference standard | Not specified in manuscript. |
| Test results | 23 | Cross tabulation of the index test results (or their distribution) by the results of the reference standard | See supplementary material 7 |
|  | 24 | Estimates of diagnostic accuracy and their precision (such as 95% CIs) | See tables 2, 3 and figure 2 |
|  | 25 | Any adverse events from performing the index test or the reference standard | Not specified in manuscript: No adverse events occurred as a result of any of the index tests. |
| **Discussion** | | | |
|  | 26 | Study limitations, including sources of potential bias, statistical uncertainty and generalizability | See discussion |
|  | 27 | Implications for practice, including the intended use and clinical role of the index test | See discussion |
| **Other information** | | | |
|  | 28 | Registration number and name of registry | The study was not registered |
|  | 29 | Where the full study protocol can be accessed | The full study protocol is not publicly available but may be requested from the corresponding author. |
|  | 30 | Sources of funding and other support; role of funders | See paragraph on funding |
